# Parvules

**Published:** 1878-01-20

**Authors:** 


PARVULES.—Wm. R. Warner & Co. have kindly presented us with samples of their *parvules*—very neatly and tastefully prepared in doses so minute that, by increasing or diminishing the number, almost any dose or fraction of a dose may be given. Even the homoepathist can use them, and no refinement of style or delicacy of taste can find room to complain of these unique and elegant preparations.

